# Comparative evaluation of otolith and fin ray as tools for assessing population differentiation in Atlantic sturgeon (*Acipenser oxyrinchus*)

**DOI:** 10.1371/journal.pone.0343989

**Published:** 2026-03-02

**Authors:** Simon Bernatchez, Louis Landry-Massicotte, Yves Paradis, Olivier Morissette, Anne-Lise Fortin, Sabrina Villeneuve, Léon L’Italien, Pascal Sirois

**Affiliations:** 1 Ministère de l’Environnement, de la Lutte contre les changements climatiques, de la Faune et des Parcs, Direction principale de l’expertise sur la faune aquatique, Québec, Québec, Canada; 2 Ministère de l’Environnement, de la Lutte contre les changements climatiques, de la Faune et des Parcs, Direction de la gestion de la faune du Bas-Saint-Laurent, Rimouski, Québec, Canada; 3 Chaire de recherche sur les espèces aquatiques exploitées, Université du Québec à Chicoutimi, Chicoutimi, Québec, Canada; 4 Ministère de l’Environnement, de la Lutte contre les changements climatiques, de la Faune et des Parcs, Direction de la gestion de la faune Capitale-Nationale-Chaudière-Appalaches, Québec, Québec, Canada; University of Messina, ITALY

## Abstract

Atlantic sturgeon (*Acipenser oxyrinchus* Mitchill, 1815) is a long-lived, anadromous species known for its extensive migrations between freshwater and marine habitats throughout its life. Despite increased conservation efforts, there is only limited information on the early life stages and habitat use of the St. Lawrence River (SLR) and Saint John River (SJR) populations in Eastern Canada. This study explores the use of otolith and fin ray microchemistry to distinguish Atlantic sturgeon populations and investigate habitat use throughout their lives. The otoliths and fin rays of fish caught in both the SLR and SJR regions were analyzed using LA-ICP-MS procedures to determine their elemental signatures throughout their lifespan (coretoedge). Using a machine learning classification approach, both otolith and fin ray revealed strong potentials for differentiating individuals from the SLR and SJR populations. Fin ray microchemistry showed a higher reclassification success and produced more contrasted elemental signatures compared to otolith microchemistry. Our results also showed that the integration of elements is dependent on the structure (i.e., fin ray or otolith). A significant correlation between element concentrations at the core of fin rays and otoliths was observed for only three elements (Sr, Mn, and Li). This phenomenon should be further investigated and considered in future applications because it could lead to reclassification errors. These findings suggest that fin ray microchemistry is a powerful approach that can be used to discriminate population origin and infer life history patterns of Atlantic sturgeon. These results are especially relevant since we propose a cost-effective method to monitor population structure and habitat use without lethal sampling. This is an essential step toward careful management of this species in Eastern Canada.

## Introduction

Atlantic sturgeon (*Acipenser oxyrinchus* Mitchill, 1815) is an anadromous fish distributed along the Atlantic coast of North America, from Newfoundland and Labrador to Florida. While the species is not legally designated as endangered in Canada, all United States populations are listed as endangered or threatened under the Endangered Species Act. Two major Atlantic sturgeon spawning populations are known in Canada, the St. Lawrence River (SLR) and the St. John River (SJR) populations. These populations are considered as designatable units by the Committee on the Status of Endangered Wildlife in Canada [1]. Both populations are commercially exploited. In the SLR (Québec), the commercial fishery targets juvenile and subadult individuals, with a maximum fork length (FL) of 1.5 m [2]. In the SJR (New Brunswick), the fishery targets subadult and adult individuals, with a minimum total length of 1.3 m [3]. Although these populations show significant genetic differentiation and seem to exhibit high spawning-river fidelity [1,4], their respective natural ranges are known to overlap: migrations from the SLR to as far as Newfoundland and Labrador in Canada and New Jersey in the United States (MELCCFP unpublished data) have been documented [5]. Considering the extent of these migrations and the legal designation of most of the populations, a cost-effective method for assessing population differentiation and habitat use could be valuable for conservation and management of Atlantic sturgeon.

Calcified structures such as scales, otoliths, and fin rays have widely been used for age and growth determination of fishes [6–14], proving their utility in fisheries sciences. Trace and minor elements are incorporated daily in the calcified structures through biomineralization, providing unique elemental fingerprints to each individual [15–17]. Otoliths, which are the most commonly used calcified structures, exhibit diverse shapes and biomineralogical compositions (aragonite, calcite, or vaterite) depending on the species. As technologies have advanced and become more accessible, analysis of the elemental composition of these structures integrated throughout the animal’s lifespan has become a powerful approach for addressing ecological questions and improving conservation and management [18,19]. In invasive species research, for example, otolith chemistry has been used to assess the migratory capacity or dispersal of species, identify natal origins, and conduct mixed‑stock assessments [20,21]. For endangered species, microchemistry has proven useful for distinguishing wild and hatchery-reared fish [22–24], identifying area of fish origin [25], assessing habitat use patterns, and identifying essential habitats [26,27].

In wild sturgeons, trace element analysis of calcified structure has focused on several topics, including natal origin, migratory and movement history, life history, habitat use, age, and age at sexual maturity [25,28–40]. Because most sturgeon species are endangered or threatened [41] and otolith sampling is lethal, only a few microchemistry studies have focused on this structure [29]. In fact, most studies have been based on the analysis of other calcified structures, such as pectoral fin rays, where sampling is nonlethal [42–45]. The removal of a section of a pectoral fin ray (fin spine) is known to heal and regenerate in sturgeons [44,46] and has shown limited impacts on swimming performance [44,45]. While dorsal scutes—the distinctive bony plates of sturgeons—have occasionally been used in sturgeon studies [34,47], fin rays are the primary structure used to study sturgeon microchemistry [25,26,28,30,31,33,35,36,38–40].

Despite substantial research efforts, few studies have directly compared multiple calcified structures, especially otoliths, for delineating sturgeon populations. The existence of commercial Atlantic sturgeon fisheries in both the SLR and SJR provides a unique opportunity to compare the power of otoliths and other structures for this purpose. Consequently, to address this gap, we evaluated: (i) the resemblance of the core elemental signatures between otoliths and fin rays in individual Atlantic sturgeon, and (ii) the reassignment accuracy of each structure based on microchemical signatures from the core and the core-to-edge transects.

## Materials and methods

### Study area and sampling

**Fig 1:**
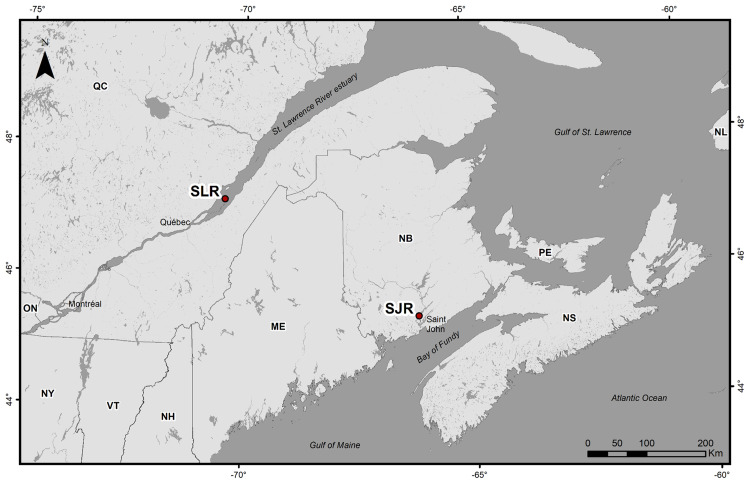
Atlantic sturgeon sampling sites. SLR: St. Lawrence River; SJR: St. John River. Sampling locations are approximate. This map was created using ArcMap 10.8.2 from esri (https://www.esri.com/en-us/home). All map shapefiles are under public domain. The basemap and boundaries shapefiles were downloaded from Open Government (https://open.canada.ca/data/en/dataset/d0cdef71-9343-46c3-b2e7-c1ded5907686/resource/51e0db22-b5ef-40b8-b260-eca19d6564fe [48]) and United States Census Bureau (https://www.census.gov/geographies/mapping-files/2017/geo/carto-boundary-file.html [49]). The hydrography shapefiles were downloaded from Open Government (https://open.canada.ca/data/en/dataset/d0cdef71-9343-46c3-b2e7-c1ded5907686/resource/51e0db22-b5ef-40b8-b260-eca19d6564fe [50]) and Données Québec (https://www.donneesquebec.ca/recherche/dataset/base-de-donnees-geographiques-et-administratives/resource/75f36728-df91-40f7-aeac-02e90d6c4d6e [51]). Contains information licensed under the Open Government Licence – Canada.

Atlantic sturgeons (heads and pectoral fins) were obtained through commercial fisheries in Eastern Canada. Fish from Québec were sampled in the SLR estuary in June 2021 (Fig 1, Table 1). Fish from New Brunswick were sampled in the SJR estuary in May 2022 (Fig 1, Table 1). All fish were caught using commercial gillnets with mesh sizes of 20.3 cm (8 in) in Québec and 33 cm (13 in) in New Brunswick, according to the regulations of each commercial fishery.

**Table 1:** Sample information for Atlantic sturgeons from the St. Lawrence River and the St. John River. Fork lengths are presented as mean ± SD (range). SLR: St. Lawrence River; SJR: St. John River.

Sampling site	n	Fork length (mm)
SLR	17	1116 ± 150 (950 − 1450)
SJR	13	1596 ± 69 (1473 − 1727)

This study exclusively utilized samples from fish captured by licensed commercial fishermen during their regular fishing operations. All specimens were already deceased and had been processed by the fishermen prior to sample collection. As no live fish were handled, sacrificed, or subjected to experimental procedures by the researchers, approval from an animal research ethics committee was not required.

### Otolith and fin ray preparation

**Fig 2:**
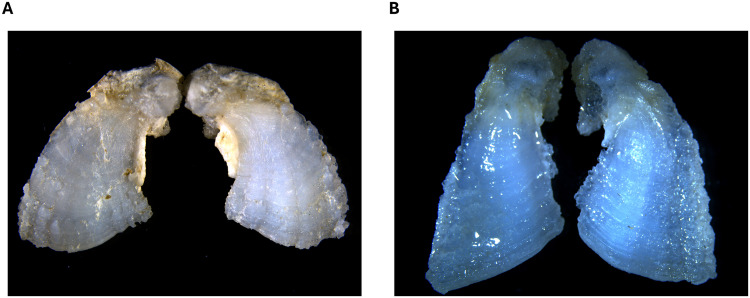
Left and right sagittal otoliths (10X) from (A) a St. Lawrence River Atlantic sturgeon and (B) a St. John River Atlantic sturgeon.

**Fig 3:**
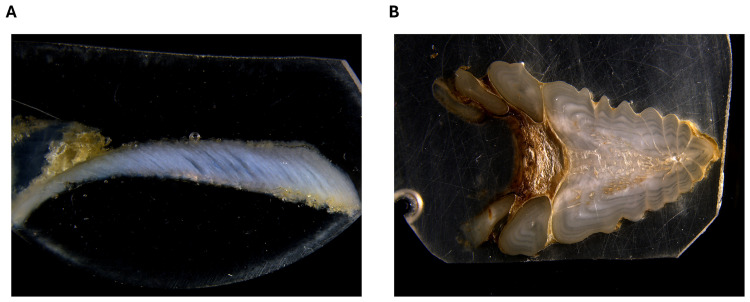
St. Lawrence River Atlantic sturgeon (A) otolith slice (16X) and (B) fin ray slice (10X).

Sagittal otoliths (Fig 2) were extracted using polytetrafluoroethylene-coated tweezers, cleaned of adhering tissues, rinsed with ultrapure water, and dried under a laminar flow cabinet for 24 hours. Each right otolith was then embedded in two-part epoxy resin (resin 100 and hardener 95; Miapoxy, Freeman, Ohio, United States) and sectioned on the traversal plane using a diamond-bladed saw (Isomet low speed saw; Buehler, Illinois, United States) to obtain 630 µm transverse sections that included the core. Sections (Fig 3a) were then sanded with sandpaper (1 200 µm; Wetordry 3M) and aluminium-oxide lapping film (5 and 1 µm; 3M), triple rinsed with ultrapure water, air dried under a laminar flow cabinet for 24 hours, and fixed on petrographic microscope slides using double-sided adhesive tape (3M).

Large sections (approximately 1.5 cm) of fin rays were cut near their base using a diamond cutting disc and a rotary tool. Each right fin ray was then embedded in two-part epoxy resin (resin 100 and hardener 95; Miapoxy, Freeman, Ohio, United States) and sliced transversally with a diamond-bladed saw (Isomet saw; Buehler, Illinois, United States) to obtain a straight cutting axis. Two 630 µm sections (Fig 3b) were then sliced and sanded using sandpaper (2 500 and 1 200 μm; Wetordry 3M) and aluminium oxide lapping films (5 and 1 μm; 3M). The best of the two (i.e., with the most visible growth marks) was fixed on petrographic microscope slides using double-sided adhesive tape (3M). Slices were not rinsed with ultrapure water to prevent collagen rehydration and warp, which occurred during preliminary tests.

### Laser Ablation-Inductively Coupled Plasma-Mass Spectrometry

**Fig 4:**
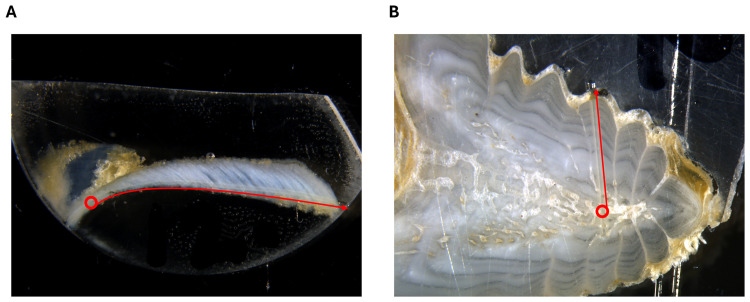
Laser ablation from an Atlantic sturgeon (A) otolith (12.5X) and (B) fin ray (20X). The red circles mark the structures’ cores. Arrows indicate the direction of the core-to-edge transects.

The Laser Ablation-Inductively Coupled Plasma-Mass Spectrometry (LA-ICP-MS) analyses were conducted using a RESOlution M-50 Excimer 193 nm laser equipped with a Laurin Technic S155 dual volume sample cell (Applied Spectra, California, United States) coupled to an Agilent model 7900 ICP-MS (Agilent, Mississauga, Ontario, Canada) located in the LabMaTer facilities (Université du Québec à Chicoutimi, Chicoutimi, Québec, Canada). Laser ablation was realized in transect mode from the core to the margin for both otoliths and fin rays (Fig 4). Laser beam diameter was set to 44 μm, speed 10 μm·s^-1^, frequency 25 Hz, laser fluence 5 J·cm^2^, and dwell time 0.252 s. A total of 38 elemental isotopes were measured (^7^Li, ^11^B, ^23^Na, ^24^Mg, ^25^Mg, ^26^Mg, ^27^Al, ^29^Si, ^31^P, ^34^S, ^39^K, ^42^Ca, ^43^Ca, ^44^Ca, ^55^Mn, ^56^Fe, ^57^Fe, ^59^Co, ^60^Ni, ^61^Ni, ^63^Cu, ^64^Zn, ^65^Cu, ^66^Zn, ^69^Ga, ^75^As, ^79^Br, ^85^Rb, ^86^Sr, ^87^Sr, ^88^Sr, ^114^Cd, ^120^Sn, ^136^Ba, ^137^Ba, ^138^Ba, ^202^Hg, and ^208^Pb). Calcium (^44^Ca) was used as an internal standard, and its concentration was normalized to 407 200 ppm in otoliths [16] and 270 000 ppm in fin rays [31]. For calibration, NIST-610 glass (NIST, United States) reference material was analyzed for 60 seconds after every 10 samples, and reference trace element concentrations were provided by Max-Planck Institute (Germany). MACS-3 and GP-4 (USGS, United States) were also analyzed at the same frequency for quality control. Laser ablation analyses were conducted over a three-day period, and the order of otolith and fin ray analyses was randomized.

Trace element concentrations were estimated using the Igor Pro Software (Wavemetrics Inc., Portland, Oregon, United States) and Iolite extension [52,53]. Data integration was performed using the “Trace elements IS” function. Trace elements limits of detection (LOD) were calculated using three times the standard deviation of the gas blank (SD_blank_), mean of each isotope count, and corrected for recovery rates of ^44^Ca. Element concentrations below LOD were excluded. Of the 38 trace elements measured, 24 were retained (^7^Li, ^11^B, ^23^Na, ^25^Mg, ^27^Al, ^29^Si, ^31^P, ^34^S, ^39^K, ^55^Mn, ^57^Fe, ^59^Co, ^61^Ni, ^64^Zn, ^65^Cu, ^69^Ga, ^75^As, ^79^Br, ^85^Rb, ^88^Sr, ^114 Cd^, ^120^Sn, ^138^Ba, and ^208^Pb). Calcium was excluded because concentrations were determined beforehand. For elements with several isotopes, only the most abundant isotope was retained. SD_blank_ (without ablation) was measured for 30 seconds before analysis and subtracted from the values. A 200 μm section (measured for 20 s) at the core of otoliths and fin rays was considered to encompass the material deposited at the natal origin site of each fish. For simplicity, mass numbers will be omitted in the following text; for example, Mn will be used instead of ⁵⁵Mn.

### Data analysis

We analyzed elemental chemistry data to compare elemental signatures between capture sites (SLR and SJR) and between structures (otoliths and fin rays). Analyses were performed on two distinct parts of each structure: the core, representing the natal origin of individuals, and the transect (from core to edge), reflecting their entire life history until capture. All data manipulation and statistical analyses were performed using R software [54].

#### Core analysis.

Core elemental concentrations were compared between structures and locations. When data were normally distributed, we used Student’s t-test (*t.test* function) to compare elemental concentrations in both otolith and fin ray cores between SLR and SJR. When data did not meet parametric assumptions, a Kruskal-Wallis test (*kruskal.test* function) was used instead. Elements that showed significant variation between sites were included in a principal component analysis (PCA; *prcomp* function) [55] to better appreciate how the elemental signatures of individuals caught in the SLR and SJR differentiate. The PCA analysis was conducted separately for otoliths and fin rays to assess how the elemental signatures varied between structures. A supervised Random Forest (*randomForest* function) [56] was used to assess reclassification success of sturgeons to their respective catch site based on core trace element concentrations and structures. Finally, correlation analyses were conducted to determine whether variations in core elemental concentrations were consistent between structures for all individuals.

### Transect analysis

To assess the reclassification capability of each structure, we used a time-series clustering analysis based on the dynamic time-warping distance (*dtwclust* package) [57] of Sr and Ba transect data (core to edge of otolith and fin ray). Sr and Ba were selected for this analysis because these elements are known to effectively discriminate saltwater and freshwater environments and are thus relevant for comparing habitat use [58]. Briefly, *dtwclust* compares two time series to find the optimal warping path between them, under certain constraints, as a way to compare their shapes. To use a multivariate clustering approach, we combined and interpolated the Sr and Ba time series to similar lengths prior to time-series comparisons and dissimilarity measurements. Otolith transects were interpolated to 1646 points whereas fin ray transects were interpolated to 618 points, and both were finally considered by relative distance (percentage of the path). Each time series was then classified by hierarchical clustering analysis according to their partition around medoids (PAM), which are average time series derived from the raw data from the groups to be classified. To find the optimal number of similar groups (i.e., clusters) within the transect data, the *clValid* [59] and *cvi* functions of the *dtwclust* package were used simultaneously, enabling the calculation of seven diagnostic indices. Potential grouping numbers between two and four were tested for both otolith and fin ray data. Once the optimal clustering configuration was selected, each Atlantic sturgeon was assigned to a group representing distinct migratory behaviour types as inferred from their combined Sr and Ba profiles.

## Results

### Core analysis

Significant differences in mean were observed between SLR and SJR otolith core elemental concentrations for B (t = −2.16; p = 0.039), Na (t = −2.74; p = 0.01), Mn (χ² = 3.95; p = 0.047), and Sr (χ² = 17.0; p < 0.001). These variations were not consistently reflected in the fin ray cores, where significant differences were observed for B (t = 3.01; p = 0.006), Mn (χ² = 16.65; p < 0.001), and Sr (χ² = 21.39; p < 0.001) but not for Na (t = 0.54; p = 0.591). Significant differences in the elemental concentrations of fin ray cores were also observed for Li (χ² = 19.87; p < 0.001), K (χ² = 6.62; p = 0.01), and Cu (χ² = 14.01; p < 0.001).

**Fig 5:**
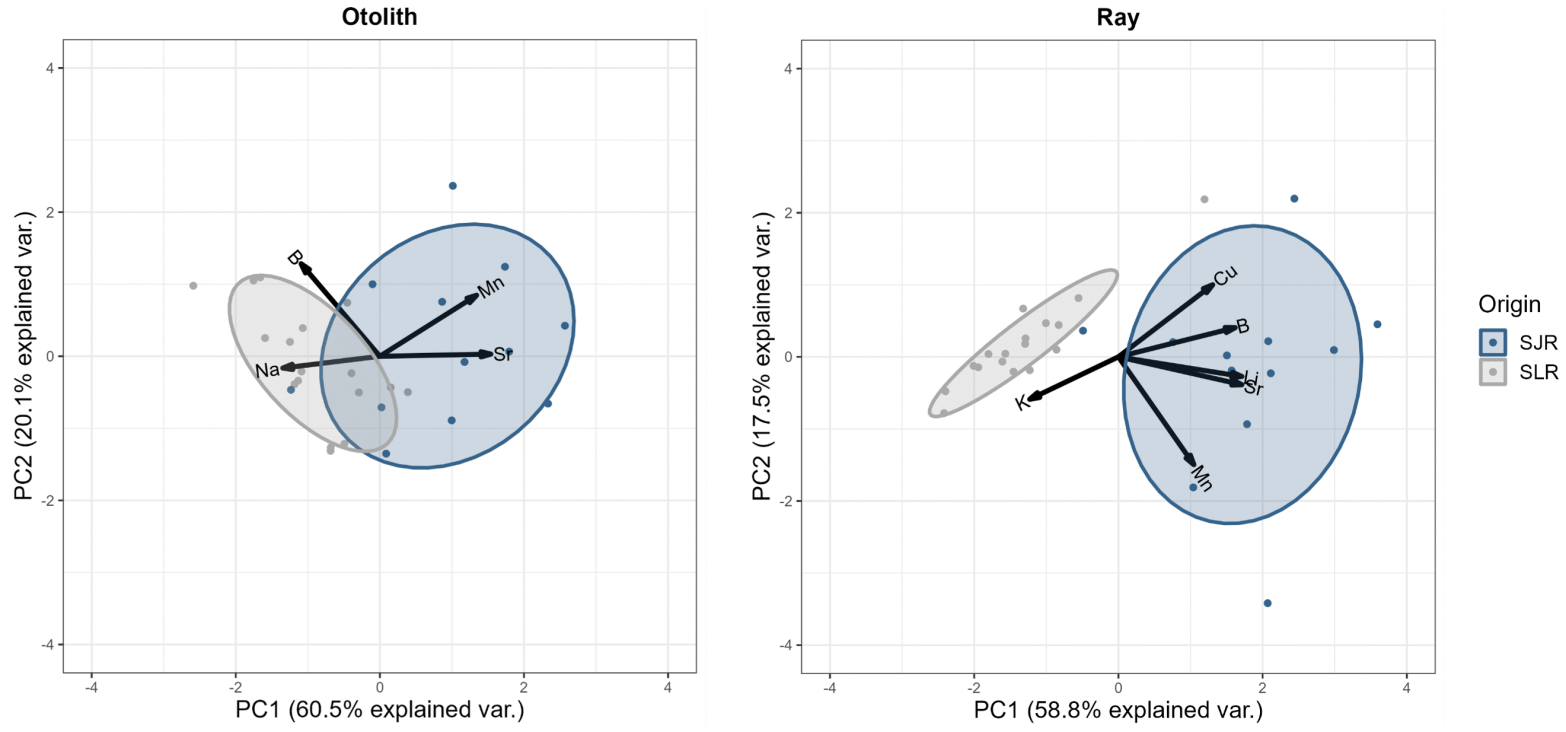
Scatterplot of a PCA comparing otolith (left panel) and fin ray (right panel) core microchemistry between St. Lawrence River (grey dots) and St. John River (blue dots) Atlantic sturgeons. A combination of B, Na, Mn, and Sr core concentrations was used to delineate place of origin elemental fingerprints for otoliths whereas a combination of Li, B, K, Mn, Cu, and Sr was used for fin rays. Ellipses correspond to approximately one standard deviation (68% confidence region) around the group centroids.

Since these elements best discriminated the elemental signature of SLR and SJR Atlantic sturgeons, their multivariate variations were subsequently explored in a PCA (Fig 5). For otolith core data, PC1 And PC2 respectively explained 60.5% and 20.1% of the total variance. Na, Mn, and Sr mainly influenced PC1 whereas B mainly influenced PC2. For fin ray core data, PC1 and PC2 explained 58.8% and 17.5%, respectively, of the total variance. The PCA results showed that the elemental signatures from fin rays differentiated SLR and SJR Atlantic sturgeons more clearly than those from otoliths. Similarly, the Random Forest analysis revealed that fin rays had a higher reclassification success than otoliths (Table 2). When using otolith microchemistry, respectively 8% of SLR and 26% of SJR individuals were misclassified to the population of origin, for a mean reclassification error of 17%. On the other hand, 10% of both SLR and SJR individuals were not correctly reassigned when using fin ray microchemistry.

**Table 2:** Random Forest reclassification matrix based on Atlantic sturgeon otolith and fin ray core microchemistry. Reclassification errors (percentages) correspond to the proportion of Atlantic sturgeons from a sampling site that have been assigned to the other sampling site based on elemental signatures from otoliths and fin rays (B, Na, Mn, and Sr for otoliths and Li, B, K, Mn, Cu, and Sr for fin rays). SLR: St. Lawrence River; SJR: St. John River.

Structure	Reclassification error (%)		
**SLR**	**SJR**	**Mean**
Otolith	8	26	17
Fin ray	10	10	10

**Fig 6:**
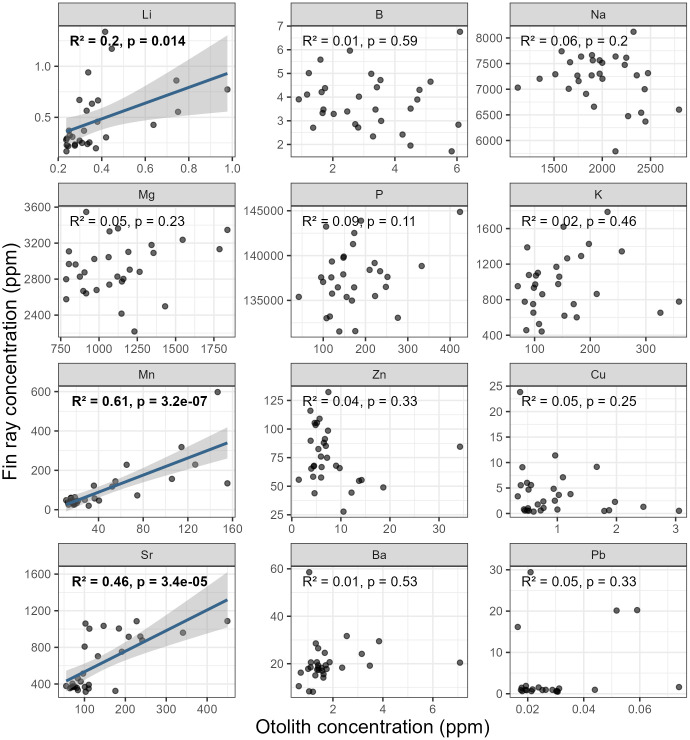
Pearson correlation showing the relationship between otolith and fin ray core microchemistry of 12 trace elements of Atlantic sturgeons originating from the St. Lawrence and St. John rivers. Significant relationships are identified in bold font.

Correlation analyses offer early insight into differences in elemental incorporation into the core regions of otoliths and fin rays. Of the 12 elements that were kept for statistical analysis, significant correlations between elemental concentrations of otolith and fin ray cores were only observed for Li, Mn, and Sr (Fig 6). Mn and Sr exhibited a strong significant relationship (respectively, R^2^ = 0.61 and R^2^ = 0.46; p < 0.001) while Li showed a moderate but still significant relationship (R^2^ = 0.20; p = 0.014). The remaining elements (B, Na, Mg, P, K, Zn, Cu, Ba, and Pb) did not exhibit significant correlations and were thus considered unrelated.

### Transect analysis

**Fig 7:**
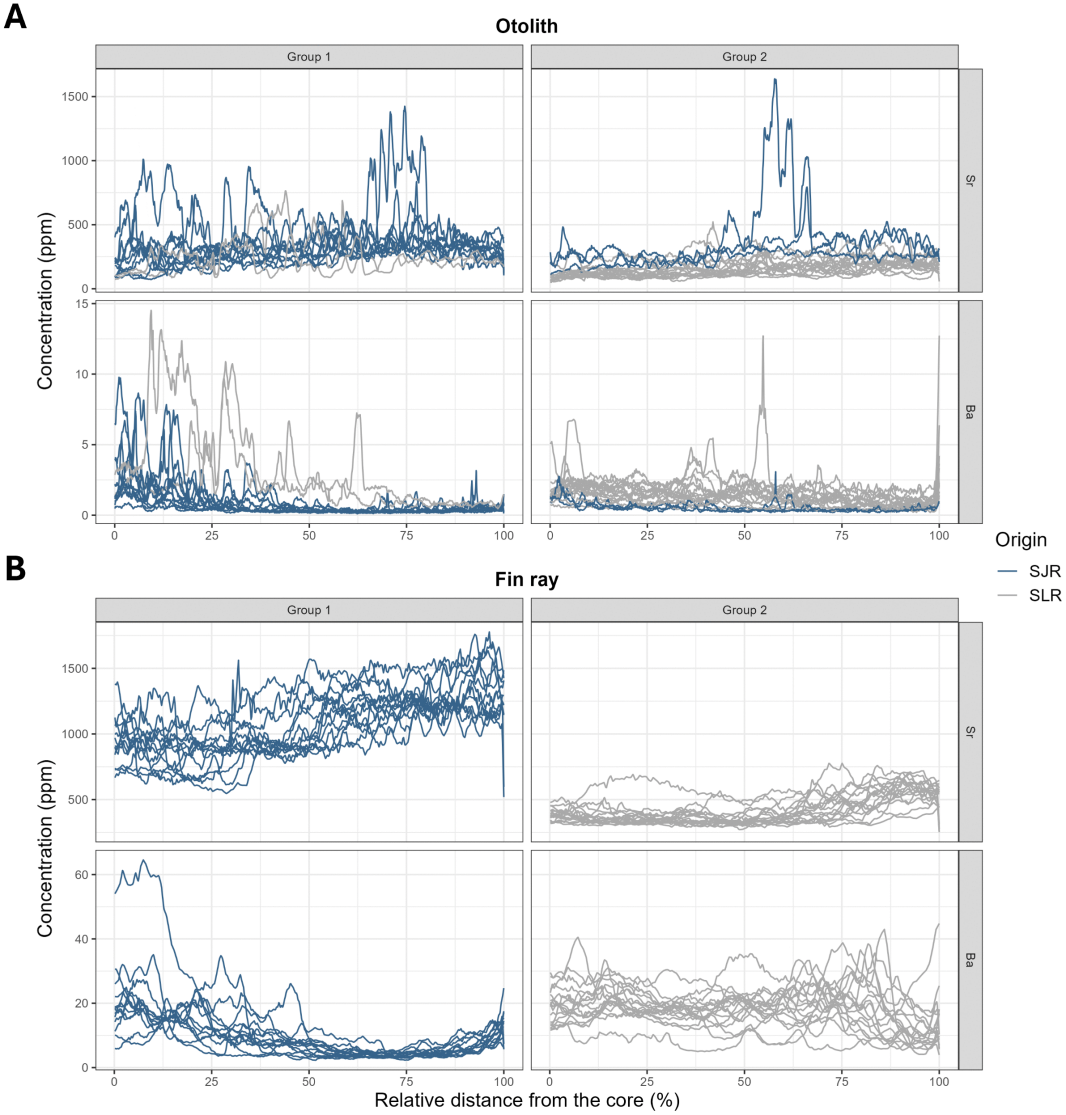
Multivariate hierarchical clustering of the Sr transects and Ba otolith (A) and fin ray (B) transects. **Both panels show the individual Sr and Ba transects, respectively, classified within both groups.** Individuals from St. Lawrence River are represented with grey lines and individuals from the St. John River are represented with blue lines.

Core-to-edge clustering analysis of otolith and fin ray microchemistry revealed two clusters (i.e., groups with similar habitat use) when using Sr and Ba data simultaneously. For otolith and fin ray analyses, group 1 showed significantly higher Sr concentrations (respectively t = 102.09, p < 0.001 and t = 237.91, p < 0.001; Fig 7). When using otolith data, group 1 had a mean Sr concentration of 309.22 ± 145.78 ppm and group 2 a mean concentration of 184.64 ± 110.66 ppm. Differences between groups were more pronounced when using fin ray data: group 1 had a mean Sr concentration of 1081.84 ± 232.44 ppm and group 2 a mean concentration of 418.54 ± 104.92 ppm. Trends in Ba concentrations were also significantly different between the two groups when using otolith and fin ray data. For the two structures, group 2 showed significantly higher concentrations (respectively t = −10.94, p < 0.001 for otoliths and t = −78.76, p < 0.001 for fin rays). Distinct value ranges were again observed between otolith and fin ray data: when using otolith data, group 1 showed a mean Ba concentration of 1.13 ± 1.66 ppm and group 2 a mean concentration of 1.27 ± 0.80 ppm, whereas group 1 had a mean Ba concentration of 9.82 ± 7.94 ppm and group 2 a mean concentration of 18.34 ± 6.37 ppm when using fin ray data.

In this study, it was assumed that Atlantic sturgeon captured in each region belonged to their respective populations (i.e., SLR and SJR). Accordingly, transect reclassification should reflect the distinct migratory behaviours of these populations. The results partly supported this expectation, with the presence of two clusters being consistent with the existence of two populations in Eastern Canada. However, like the core data analysis, misclassifications occurred when using otolith transect data. Two individuals captured in the SLR were reassigned to group 1, which was composed mainly of SJR individuals (10 of 13; Fig 7A), while three SJR individuals were reassigned to group 2, which was dominated by SLR fish (15 of 17; Fig 7A). These errors correspond to a 17% misclassification rate for population reassignment using otolith transects. In contrast, fin ray transect data provided stronger discrimination, correctly reassigning all 13 SJR individuals to group 1 and all 17 SLR individuals to group 2 (Fig 7B). These findings indicate that fin ray microchemistry transects have a greater potential than otolith transects for accurate population reassignment.

## Discussion

The analysis of otoliths and fin rays using a machine learning classification approach revealed significant potential for accurate population discrimination in Atlantic sturgeon. Compared to otoliths, population reclassification success was higher with fin ray core analysis and allowed better discrimination of elemental signatures.

According to our results, there appears to be a mismatch in the integration of elements in otoliths and fin rays. Indeed, we observed a significant correlation between element concentrations in the core of both structures for only three of the 12 retained elements. Previous research found that the otolith composition of sturgeons and paddlefishes (order Acipenseriformes) was heterogenous within and between otoliths, with the coexistence of CaCo_3_ polymorphs (vaterite and calcite) among and within otoliths being the plausible cause [60]. It is known that calcium carbonate polymorphs incorporate trace elements at different rates [61], likely linked to the difference in their calcium density, which could be influenced by ontogenetic development and temperature [60,62]. This is especially important when comparing different structures or with heterogeneous structures because calcium content is assumed to be constant in the analytical procession of LA-ICP-MS data. Hence, a polymorph-specific calibration may be needed when expressing elemental ratios to calcium to avoid bias in the interpretation of migratory history or habitat use patterns [60,63]. Because calcium concentration was assumed to be constant for all otoliths in the present study, this may partially explain why correlation was found for only 25% of the elements retained for the analyses and for the more pronounced differences observed with this structure.

Additionally, some previous studies have documented the existence of resorption and elemental reworking phenomenon in the fin rays of sturgeon and other fish species, probably more in the early growth bands [64]. However, the true magnitude of this reworking remains unknown. Some authors have suggested that this process may be related to nutritional stress episodes, acting as a calcium reservoir analog to bones [65]. Hence, variable elemental reworking depending on nutritional states or environmental conditions may partially explain why the combination of elements that best discriminated individuals from the two rivers was different among structures. Another remaining challenge is the temporal pairing of structures. Ingram et al. [47] showed how relative distance between growth annuli may vary between structures and generate variations in the timing and the nature of trace element integration. Identifying these annuli in the structures used in the current study may have allowed better interpretation of the results and led to a better temporal assessment of habitat use. However, growth annuli were often unclear and difficult to distinguish since they tended to overlap. Because otoliths are much smaller than fin rays, this could lead to lower resolution and thus less reliable results, especially in long-lived Atlantic sturgeon.

Results from the multivariate approach (PCA) using the fin ray data suggest that two fish assumed to originate from one river were harvested in the other. Thus, these fish could have been considered migrants from one system to the other. Previous capture–mark–recapture and ongoing acoustic telemetry studies documented Atlantic sturgeons moving from the SLR to as far as Newfoundland and Labrador in Canada [5] and New Jersey in the United States (MELCCFP unpublished data). Considering the extent of these movements, among-system migrants are plausible. However, results from the transect analysis suggest that all fish caught in a river have similar habitat-use patterns in terms of Sr and Ba concentrations. Knowing that SLR and SJR are several hundred kilometres apart, and that juvenile Atlantic sturgeons stay in fresh or brackish waters for at least a couple years [1,66], it seems unlikely that the signatures of these two individuals would still group with the other individuals from their native river in the transect analysis. Moreover, Sr concentrations in the first 5–10% of the relative distance from the core do not seem to overlap between individuals harvested in the SLR and SJR. Results from the PCA using otolith data are less clear and could have been biased by many factors including different calcium polymorphs, the normalization of calcium concentration for the entire otolith, or maternal effect. Furthermore, none of the putative migrants suggested by the PCA results using fin ray data matches the fish that could be identified as migrants by the transect analysis conducted on otolith data.

The transect analysis showed that SLR sturgeons were predominantly associated with brackish waters in comparison with sturgeons from the SJR, which were mainly associated with salty waters. In fact, calcified structures of the SLR sturgeons were characterized by lower concentrations of Sr and Ba, an indication of freshwater environments [17]. These results are consistent with the known ecology of the SLR Atlantic sturgeon. The SLR is a large river draining the Great Lakes, which is one of the largest groups of freshwater lakes on Earth. The SLR estuary is divided in three sections, a fluvial estuary, a middle estuary, and a maritime estuary. The middle estuary, where surface-layer salinity ranges from ~2–24 ‰ [67], is known to be an important zone where juvenile and subadult Atlantic sturgeons aggregate and feed [68–71]. However, as the SJR is considerably smaller than the SLR, salinity in the SJR estuary rapidly increases as the freshwater reaches the Bay of Fundy, which is directly connected to the Atlantic Ocean. These results indicate that—based on core-to-edge profiles of fin ray Sr and Ba—SLR and SJR individuals exhibited distinct habitat use, which could be useful in distinguishing populations.

All things considered, fin rays offer several advantages over otoliths. Fin rays showed a higher reclassification success and clearer patterns of inferred habitat use. Because fin ray removal is nonlethal and this structure is already widely used to estimate age in sturgeons [37,72–75], these results are especially interesting for the prudent management and conservation of Atlantic sturgeon. In fact, use of a reliable nonlethal structure is mandatory for most sturgeon populations. Otolith removal necessarily implies killing fish and thus cannot be used for unexploited populations. This significantly limits its use for any type of analysis in sturgeon. Our study supports using the fin ray as the preferred calcified structure for sturgeon aging and microchemistry analyses.

In comparison with other technologies, microchemistry offers several advantages. Compared to genomics (and genetics), it can be cheaper and can address ecological questions in addition to population assignment that genomics cannot. In fact, if prior knowledge on trace-element concentrations in the studied system is available, microchemistry can be used to identify the origin and migratory history of a fish within a system [20,76,77]. Moreover, unlike acoustic telemetry, there is no need to deploy a network of receivers and wait until the data are generated in real time, which represents a considerable amount of work and is a long-term process. Using microchemistry, researchers examine conditions that were present before a fish was caught. However, the resolution for tracking movements is considerably lower than acoustic telemetry. Microchemistry should be used to answer ecological questions requiring this lower level of resolution, such as natal river identification, general habitat use, and migration ontogeny.

Although the present study suggests that fin ray analysis can be a reliable tool for sturgeon conservation and management, there are still some limits and caveats that must be acknowledged. First, the number of fish in this study is relatively low. Including more fish could have led to more reliable reclassification results or showed an unexpected variability. Moreover, to corroborate the origin of each fish in the present study, an individual-based assignment test could have been conducted using the microsatellite genetic baseline for North American Atlantic sturgeon described in White et al. [4]. This analysis could have helped us to interpret and qualify results by considering the possibility of migrants.

Despite these limits and caveats, the present study underlines the potential of fin ray microchemistry in sturgeon research, conservation, and management. In the SLR, there is a lack of knowledge regarding Atlantic sturgeon spawning [78–80], and little is known about aggregation areas of young stages [68,70,71]. It is assumed that Atlantic sturgeon spawn at multiple sites, although no eggs or larvae have ever been caught in the system, despite significant efforts by the government of Québec over the years (MELCCFP unpublished data). Identifying and locating spawning grounds and young-of-the-year aggregation sites is crucial to ensure the species’ protection and conservation. Future analyses, including a larger number of Atlantic sturgeons originating exclusively from the SLR, are needed. If more than one cluster could be identified using fin ray core data, it would suggest the presence of multiple spawning sites within the system. This approach has proved useful for other species in the St. Lawrence River system [81–83]. In combination with the knowledge acquired through several decades using acoustic telemetry [78–80], the elemental fingerprint reference database of the SLR and its tributaries that is now available [76] could help to identify spawning grounds and young-of-the-year aggregation sites and also assess their importance.
